# *catena*-Poly[[gallium(III)-bis­[μ-D/l-tartrato(2−)]-gallium(III)-di-μ-hydroxido] dihydrate]

**DOI:** 10.1107/S1600536812028188

**Published:** 2012-06-30

**Authors:** Xiaojing Liu, Ruijing Tian, Cailing Zhang, Xia Zhi, Qinhe Pan

**Affiliations:** a Department of Materials and Chemical Engineering, Ministry of Education, Key Laboratory of Advanced Materials of Tropical Island Resources, Hainan University, Haikou 570228, People’s Republic of China

## Abstract

In the title compound, {[Ga_2_(C_4_H_4_O_6_)_2_(OH)_2_]·2H_2_O}_*n*_, the Ga^III^ atom is located on a twofold rotation axis and is six-coordinated by two O atoms from bridging hydroxide groups and four O atoms from two symmetry-related tartrate units in a slightly distorted octa­hedral environment. Each tartrate unit binds to two Ga^III^ atoms as a bis-chelating bridging ligand by two pairs of hydroxide groups and an O atom of a carboxyl­ate group. The Ga^III^ atoms are linked by two bridging hydroxide groups located on mirror planes. In this way a chain along the *c* axis is formed. Free water mol­ecules on mirror planes are located between the chains and hold them together through hydrogen-bonding inter­actions, with O⋯O distances in the range 2.509 (3)–3.179 (5) Å.

## Related literature

For the potential applications of coordination polymers in drug delivery, shape-selective sorption/separation and catalysis, see: Chen & Tong (2007 ▶); Zeng *et al.* (2009 ▶). For a description of their one-dimensional to three-dimensional architectures, see: Du & Bu (2009 ▶); Qiu & Zhu (2009 ▶). For our recent research on the synthesis of coordination polymers, see: Pan, Cheng & Bu (2010 ▶, 2011 ▶); Pan, Cheng & Hu (2010 ▶); Pan, Li *et al.* (2010 ▶); Pan, Ma *et al.* (2012 ▶); Wu *et al.* (2011 ▶).
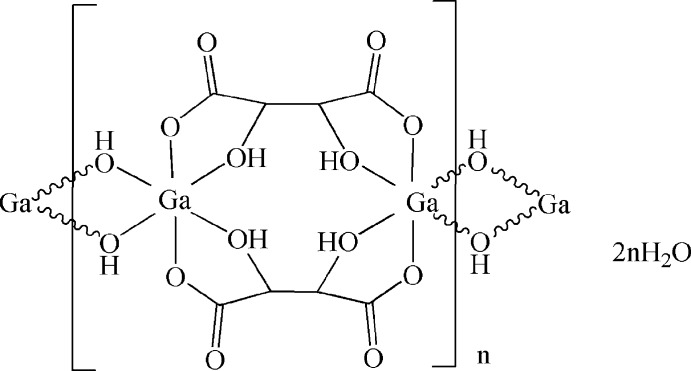


## Experimental

### 

#### Crystal data

[Ga_2_(C_4_H_4_O_6_)_2_(OH)_2_]·2H_2_O
*M* *_r_* = 505.64
Orthorhombic, 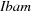

*a* = 8.6830 (17) Å
*b* = 10.797 (2) Å
*c* = 16.158 (3) Å
*V* = 1514.8 (5) Å^3^
*Z* = 4
Mo *K*α radiation
μ = 3.65 mm^−1^
*T* = 293 K
0.30 × 0.20 × 0.15 mm

#### Data collection

Rigaku R-AXIS RAPID-S diffractometer
Absorption correction: multi-scan (*CrystalClear*; Rigaku/MSC, 2002 ▶) *T*_min_ = 0.421, *T*_max_ = 0.578
7524 measured reflections
897 independent reflections
756 reflections with *I* > 2σ(*I*)
*R*_int_ = 0.045

#### Refinement

*R*[*F* ^2^ > 2σ(*F* ^2^)] = 0.031
*wR*(*F* ^2^) = 0.074
*S* = 1.14
897 reflections
63 parameters
H-atom parameters constrained
Δρ_max_ = 0.48 e Å^−3^
Δρ_min_ = −0.41 e Å^−3^

### 

Data collection: *RAPID-AUTO* (Rigaku, 1998 ▶); cell refinement: *RAPID-AUTO*; data reduction: *CrystalStructure* (Rigaku/MSC, 2002 ▶); program(s) used to solve structure: *SHELXS97* (Sheldrick, 2008 ▶); program(s) used to refine structure: *SHELXL97* (Sheldrick, 2008 ▶); molecular graphics: *SHELXTL* (Sheldrick, 2008 ▶); software used to prepare material for publication: *SHELXTL*.

## Supplementary Material

Crystal structure: contains datablock(s) I, global. DOI: 10.1107/S1600536812028188/vn2042sup1.cif

Structure factors: contains datablock(s) I. DOI: 10.1107/S1600536812028188/vn2042Isup2.hkl

Additional supplementary materials:  crystallographic information; 3D view; checkCIF report

## Figures and Tables

**Table 1:** Selected bond lengths (Å)

Ga1—O1	2.0102 (19)
Ga1—O2	1.970 (2)
Ga1—O4	1.9219 (18)

**Table 2:** Hydrogen-bond geometry (Å, °)

*D*—H⋯*A*	*D*—H	H⋯*A*	*D*⋯*A*	*D*—H⋯*A*
O1—H2⋯O3^i^	0.89	1.64	2.509 (3)	163
O4—H4⋯O1*W* ^ii^	0.89	1.91	2.774 (5)	162
O1*W*—H1*W*⋯O2	0.89	2.56	3.179 (5)	127

Symmetry codes: (i) 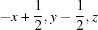; (ii) 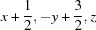.

**Fig. 1.**
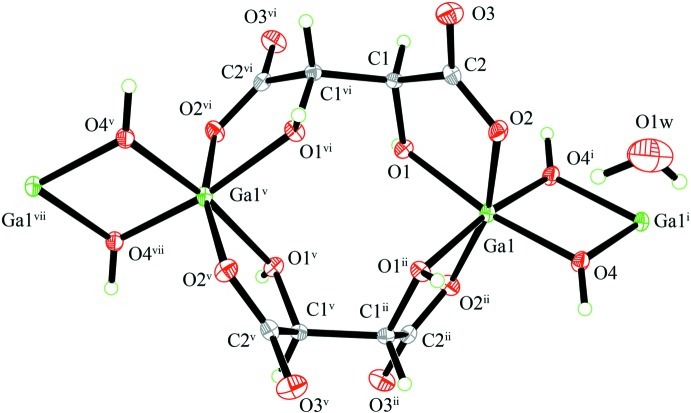
A view of the asymmetric unit of complex. Ellipsoids are drawn at the 30% probability level. [Symmetry codes: (i) 1 - x,1 - y,1 - z; (ii) 1 - x,1 - y,z; (v) x,1 - y,3/2 - z; (vi) 1 - x,y,3/2 - z; (vii) x,1 - y,1/2 + z.

## supplementary crystallographic information

### Comment

Recently, increasing attention has been paid to the design and synthesis of coordination polymers, because of their potential applications in drug delivery, shape-selective sorption/separation, and catalysis (Chen & Tong, 2007; Zeng *et al.*, 2009). Their structures vary from one-dimensional to three-dimensional architectures (Qiu & Zhu, 2009; Du & Bu, 2009). Our recent research interest has been focused on the synthesis of novel coordination polymers (Pan, Cheng & Bu, 2010, 2011; Pan, Cheng & Hu, 2010; Pan, Li *et al.*, 2010; Pan, Ma *et al.*, 2012; Wu *et al.* 2011). Here we present a Ga-containing coordination polymer with a one-dimensional chain structure.

As shown in Fig. 1, the asymmetric part of crystal structure of the title compound consists of half a Ga^III^ atom, half a tartrate anion, half a bridging hydroxide group, and half a free water molecule. The Ga^III^ atom is located on a twofold rotation axis and is six-coordinated by two O atoms from the bridging hydroxide groups, and four O atoms from two different tartrate units in a slightly distorted octahedral environment, with Ga—O bond distances in the range of 1.9219 (18) to 2.0102 (19) Å. The carboxylate groups of the tartrate are completely deprononated, the hydroxide group, however, is not. Chelated by the O1 atom from a hydroxide group and the O2 atom of neighboring carboxylate groups, each tartrate unit binds to two Ga^III^ atoms as a bis-chelating bridging ligand. Two Ga^III^ atoms and two tartrate units are linked to form a building unit, each building unit being surround by four O atoms of four different hydroxide groups, which is located on mirror, and linked two building units as the bridging hydroxide groups. In this way a one-dimensional chain along the *c* axis is formed by linking building units and the bridging hydroxide groups. Free water molecules reside between the chains while connecting them by hydrogen-bonding interactions as to form a three-dimensional supermolecular structure with O···O distances ranging from 2.509 (3)–3.179 (5) Å.

### Experimental

In a typical synthesis, a mixture of Ga_2_O_3_ (0.047 g), D,*L*-tartaric acid (0.075 g), and H_2_O (10 ml), was added to a 20 ml Teflon-lined reactor under autogenous pressure at 160 °C for 3 days, after which colorless prismatic shaped crystals were obtained.

### Refinement

All H atoms were positioned geometrically (O—H = 0.89 Å, C—H = 0.98 Å) and allowed to ride on their parent atoms, with *U*_iso_(H) = 1.2*U*_eq_(parent atom).

### Crystal data

**Table:**

[Ga_2_(C_4_H_4_O_6_)_2_(OH)_2_]·2H_2_O	*F*(000) = 1008
*M**_r_* = 505.64	*D*_x_ = 2.217 Mg m^−^^3^
Orthorhombic, *I**b**a**m*	Mo *K*α radiation, λ = 0.71073 Å
Hall symbol: -I22c	Cell parameters from 7524 reflections
*a* = 8.6830 (17) Å	θ = 3.0–27.4°
*b* = 10.797 (2) Å	µ = 3.65 mm^−^^1^
*c* = 16.158 (3) Å	*T* = 293 K
*V* = 1514.8 (5) Å^3^	Prism, colorless
*Z* = 4	0.3 × 0.2 × 0.15 mm

### Data collection

**Table:**

Rigaku R-AXIS RAPID-S diffractometer	897 independent reflections
Radiation source: fine-focus sealed tube	756 reflections with *I* > 2σ(*I*)
Graphite monochromator	*R*_int_ = 0.045
ω scans	θ_max_ = 27.4°, θ_min_ = 3.0°
Absorption correction: multi-scan (*CrystalClear*; Rigaku/MSC, 2002)	*h* = −11→11
*T*_min_ = 0.421, *T*_max_ = 0.578	*k* = −13→14
7524 measured reflections	*l* = −20→20

### Refinement

**Table:**

Refinement on *F*^2^	Primary atom site location: structure-invariant direct methods
Least-squares matrix: full	Secondary atom site location: difference Fourier map
*R*[*F*^2^ > 2σ(*F*^2^)] = 0.031	Hydrogen site location: inferred from neighbouring sites
*wR*(*F*^2^) = 0.074	H-atom parameters constrained
*S* = 1.14	*w* = 1/[σ^2^(*F*_o_^2^) + (0.0342*P*)^2^ + 2.6518*P*] where *P* = (*F*_o_^2^ + 2*F*_c_^2^)/3
897 reflections	(Δ/σ)_max_ = 0.001
63 parameters	Δρ_max_ = 0.48 e Å^−^^3^
0 restraints	Δρ_min_ = −0.41 e Å^−^^3^

### Special details

**Table:**

Geometry. All e.s.d.'s (except the e.s.d. in the dihedral angle between two l.s. planes) are estimated using the full covariance matrix. The cell e.s.d.'s are taken into account individually in the estimation of e.s.d.'s in distances, angles and torsion angles; correlations between e.s.d.'s in cell parameters are only used when they are defined by crystal symmetry. An approximate (isotropic) treatment of cell e.s.d.'s is used for estimating e.s.d.'s involving l.s. planes.
Refinement. Refinement of *F*^2^ against ALL reflections. The weighted *R*-factor *wR* and goodness of fit *S* are based on *F*^2^, conventional *R*-factors *R* are based on *F*, with *F* set to zero for negative *F*^2^. The threshold expression of *F*^2^ > σ(*F*^2^) is used only for calculating *R*-factors(gt) *etc*. and is not relevant to the choice of reflections for refinement. *R*-factors based on *F*^2^ are statistically about twice as large as those based on *F*, and *R*- factors based on ALL data will be even larger.

### Fractional atomic coordinates and isotropic or equivalent isotropic displacement parameters (Å^2^)

**Table:**

	*x*	*y*	*z*	*U*_iso_*/*U*_eq_	
Ga1	0.5000	0.5000	0.59085 (2)	0.01685 (16)	
O1	0.3677 (2)	0.42047 (17)	0.67803 (11)	0.0176 (4)	
H2	0.3488	0.3398	0.6730	0.021*	
O2	0.3489 (2)	0.63361 (18)	0.60822 (12)	0.0217 (4)	
O3	0.1354 (2)	0.68818 (19)	0.67612 (14)	0.0290 (5)	
O4	0.5962 (3)	0.5850 (3)	0.5000	0.0205 (6)	
H4	0.6917	0.6148	0.5000	0.025*	
C1	0.2355 (3)	0.4887 (2)	0.70276 (16)	0.0155 (5)	
H1	0.1423	0.4434	0.6870	0.019*	
C2	0.2388 (3)	0.6141 (3)	0.65840 (16)	0.0180 (6)	
O1W	0.4116 (5)	0.8743 (5)	0.5000	0.0808 (15)	
H1W	0.4511	0.8331	0.5428	0.097*	

### Atomic displacement parameters (Å^2^)

**Table:**

	*U*^11^	*U*^22^	*U*^33^	*U*^12^	*U*^13^	*U*^23^
Ga1	0.0208 (2)	0.0157 (2)	0.0140 (2)	−0.00133 (18)	0.000	0.000
O1	0.0212 (10)	0.0105 (9)	0.0212 (9)	0.0016 (8)	0.0037 (8)	0.0014 (8)
O2	0.0252 (11)	0.0174 (10)	0.0227 (10)	0.0019 (9)	0.0042 (9)	0.0071 (8)
O3	0.0280 (11)	0.0176 (10)	0.0414 (13)	0.0065 (9)	0.0058 (11)	0.0068 (9)
O4	0.0218 (15)	0.0234 (14)	0.0164 (13)	−0.0093 (12)	0.000	0.000
C1	0.0146 (12)	0.0139 (12)	0.0180 (13)	−0.0004 (11)	−0.0003 (11)	0.0009 (11)
C2	0.0211 (14)	0.0148 (13)	0.0180 (13)	0.0005 (11)	−0.0037 (12)	−0.0003 (10)
O1W	0.048 (3)	0.105 (4)	0.089 (4)	0.008 (3)	0.000	0.000

### Geometric parameters (Å, º)

**Table:**

Ga1—O1	2.0102 (19)	O2—C2	1.271 (3)
Ga1—O2	1.970 (2)	O3—C2	1.236 (3)
Ga1—O4^i^	1.9219 (18)	O4—Ga1^i^	1.9219 (17)
Ga1—O4	1.9219 (18)	O4—H4	0.8900
Ga1—O2^ii^	1.970 (2)	C1—C2	1.532 (4)
Ga1—O1^ii^	2.0102 (19)	C1—C1^iii^	1.546 (5)
Ga1—Ga1^i^	2.9358 (10)	C1—H1	0.9800
O1—C1	1.421 (3)	O1W—H1W	0.8900
O1—H2	0.8900		
			
O4^i^—Ga1—O4	80.41 (12)	O1^ii^—Ga1—Ga1^i^	134.49 (6)
O4^i^—Ga1—O2^ii^	92.78 (10)	O1—Ga1—Ga1^i^	134.49 (6)
O4—Ga1—O2^ii^	99.74 (10)	C1—O1—Ga1	115.90 (15)
O4^i^—Ga1—O2	99.74 (10)	C1—O1—H2	112.6
O4—Ga1—O2	92.78 (10)	Ga1—O1—H2	117.4
O2^ii^—Ga1—O2	163.61 (11)	C2—O2—Ga1	118.06 (17)
O4^i^—Ga1—O1^ii^	170.89 (9)	Ga1^i^—O4—Ga1	99.59 (12)
O4—Ga1—O1^ii^	94.76 (8)	Ga1^i^—O4—H4	125.3
O2^ii^—Ga1—O1^ii^	80.36 (7)	Ga1—O4—H4	125.3
O2—Ga1—O1^ii^	88.15 (8)	O1—C1—C2	108.2 (2)
O4^i^—Ga1—O1	94.76 (8)	O1—C1—C1^iii^	111.05 (17)
O4—Ga1—O1	170.89 (9)	C2—C1—C1^iii^	108.8 (3)
O2^ii^—Ga1—O1	88.15 (8)	O1—C1—H1	109.6
O2—Ga1—O1	80.36 (7)	C2—C1—H1	109.6
O1^ii^—Ga1—O1	91.02 (11)	C1^iii^—C1—H1	109.6
O4^i^—Ga1—Ga1^i^	40.20 (6)	O3—C2—O2	125.9 (3)
O4—Ga1—Ga1^i^	40.20 (6)	O3—C2—C1	116.7 (2)
O2^ii^—Ga1—Ga1^i^	98.19 (6)	O2—C2—C1	117.3 (2)
O2—Ga1—Ga1^i^	98.19 (6)		

Symmetry codes: (i) −*x*+1, −*y*+1, −*z*+1; (ii) −*x*+1, −*y*+1, *z*; (iii) *x*, −*y*+1, −*z*+3/2.

### Hydrogen-bond geometry (Å, º)

**Table:**

*D*—H···*A*	*D*—H	H···*A*	*D*···*A*	*D*—H···*A*
O1—H2···O3^iv^	0.89	1.64	2.509 (3)	163
O4—H4···O1*W*^v^	0.89	1.91	2.774 (5)	162
O1*W*—H1*W*···O2	0.89	2.56	3.179 (5)	127

Symmetry codes: (iv) −*x*+1/2, *y*−1/2, *z*; (v) *x*+1/2, −*y*+3/2, *z*.
